# Galectins Differentially Regulate the Surface Glycosylation of Human Monocytes

**DOI:** 10.3390/biom12091168

**Published:** 2022-08-23

**Authors:** Dina B. AbuSamra, Rafael Martínez-Carrasco, Pablo Argüeso

**Affiliations:** Tufts Medical Center, Department of Ophthalmology, Tufts University School of Medicine, Boston, MA 02111, USA

**Keywords:** galectins, inflammation, lectin, monocyte, surface glycosylation

## Abstract

Monocytes are circulating blood cells that rapidly mobilize to inflamed sites where they serve diverse effector functions shaped in part by microenvironmental cues. The establishment of specific glycosylation patterns on the immune cell glycocalyx is fundamental to direct the inflammatory response, but relatively little is known about the mechanisms whereby the microenvironment controls this process. Here, we report that galectins differentially participate in remodeling the surface glycosylation of human primary CD14^+^CD16^−^ monocytes under proinflammatory conditions. Using a lectin array on biotinylated protein, we found that the prototypic galectin-1 negatively influenced the expression of galactose epitopes on the surface of monocytic cells. On the other hand, the tandem-repeat galectin-8 and, to a certain extent, the chimeric galectin-3 promoted the expression of these residues. Jacalin flow cytometry and pull-down experiments further demonstrated that galectin-8 causes a profound upregulation of mucin-type O-glycosylation in cell surface proteins from primary monocytes and THP-1 cells. Overall, these results highlight the emerging role of the galectin signature on inflamed tissues and provide new insights into the contribution of extracellular galectins to the composition of the glycocalyx in human monocytes.

## 1. Introduction

Monocytes are a heterogeneous population of innate immune cells generated in the bone marrow and released into the bloodstream, where they constitute approximately 10% of the peripheral leukocytes [1]. Variation in the amounts of monocytes has been observed in the blood of patients with numerous pathological states, such as infections, autoimmunity, respiratory and cardiovascular diseases and inflammatory disorders [2]. Based on the expression of superficial CD14, a cell co-receptor for lipopolysaccharide, and CD16, the low-affinity IgG receptor, monocytes can be divided into three subsets: classical, intermediate, and non-classical. Classical monocytes, often called “inflammatory monocytes”, constitute about 90% of the total monocytic population and have high expression of CD14 but not CD16. These monocytes are selectively trafficked into sites of inflammation, produce inflammatory cytokines, differentiate into macrophages and dendritic cells, and play pivotal roles in providing pro-inflammatory or resolving activities [1,3]. A suitable microenvironment, driven by multiple chemical and biophysical signals, is necessary to trigger monocyte recruitment and differentiation as these cells move out of blood vessels into areas of tissue damage [4,5].

A growing body of data indicate that protein glycosylation plays an essential role in monocytic differentiation and ability to mediate cellular interactions. The expression profile of genes associated with glycosylation changes as these cells undergo differentiation into dendritic cells and macrophages [6]. Acquiring specific N-linked and O-linked glycans on the cell surface of monocytes has been shown to (i) modulate recruitment into sites of injury, (ii) facilitate communication with the adaptive immune system, and (iii) mediate host–pathogen recognition and clearance. Examples include the initiation of mucin-type O-glycosylation in monocytes, which promotes adhesion to vascular endothelial cells in atherosclerosis [7]. In addition, human monocyte-derived dendritic cells show a profound upregulation of glycan epitopes during maturation. These epitopes can be recognized by galectins and siglecs and, therefore, facilitate T cell activation [8]. Lastly, macrophage differentiation has been linked to alterations in cell surface glycosylation and, consequently, to the distinct interaction with bacterial lectins [9]. Despite these advances, our knowledge about the influence of the microenvironment in shaping the cell surface glycocalyx of monocytes remains limited.

Galectins are a family of small soluble multivalent proteins with affinity towards glycoconjugates containing β-galactose. According to structure, they can be classified into (i) prototypical, with a single carbohydrate-recognition domain that may associate to form homodimers, (ii) chimeric, with a single carbohydrate-recognition domain and a large non-lectin N-terminal domain that contributes to self-aggregation and, (iii) tandem-repeat, with two distinct but homologous carbohydrate-recognition domains in a single polypeptide [10]. Each galectin has unique glycan binding preferences that impact its engagement with cell surface receptors, thereby leading to a unique range of signaling processes [11]. Inflammatory disorders are frequently associated with the expression of unique combinations of galectins, known as the galectin signature, that define the local microenvironment. While some galectins are made by immune cells, others are secreted by different cell types, such as endothelial or epithelial cells, and bind to immune cells to regulate many aspects of the immune response [12]. The present study aimed to investigate the role of the different types of galectins in regulating the surface glycosylation of classical human monocytes under proinflammatory conditions. Using a lectin array on biotinylated protein, we found that galectins differentially influence the expression of galactose epitopes on the surface of monocytic cells. We further demonstrated that galectin-8 causes a profound upregulation of mucin-type O-glycosylation in cell surface proteins from primary monocytes and THP-1 cells, which may have important implications for the activities of monocytes during inflammation.

## 2. Materials and Methods

### 2.1. Cells and Cell Line

Leukocyte concentrates (buffy coats) from healthy donor volunteers were obtained from the Blood Transfusion Service at Massachusetts General Hospital with approval from the institutional review board (IRB protocol no. 2019P000585). Peripheral blood mononuclear cells were freshly isolated from the buffy coats by Ficoll-Paque PREMIUM (1.073 g/mL, Sigma-Aldrich, St. Louis, MO, USA) followed by erythrocyte lysis using an ice-cold NH4Cl solution for 10 min. Monocyte (CD14^+^CD16^−^) negative selection was performed via magnetic-activated cell sorting using the Classical Monocyte Isolation Kit (Miltenyi Biotec, Bergisch Gladbach, Germany) according to the manufacturer’s instructions. The human monocytes were flushed with PBS containing 2.5% human serum albumin (HSA) and 2.5 mM EDTA. This yielded >75% monocytes, as determined by flow cytometry following labeling with anti-CD14-FITC mAb (BD Biosciences, San Jose, CA, USA).

The human THP-1 monocytic cell line was cultured in RPMI 1640 medium (Life Technologies, Carlsbad, CA, USA) supplemented with 10% fetal bovine serum (Gibco, Grand Island, NY, USA), 50 μM β-mercaptoethanol and 100 units/mL penicillin-streptomycin (Invitrogen, Waltham, MA, USA). Cells were plated and maintained at a density of 2.5 × 10^5^ cells/mL by evaluating the number of cells every 2 days.

### 2.2. Expression, Purification and Treatment with Human Galectins

Expression vectors for human galectin-1, -3 and -8 were transformed into Rosetta cells as previously described [13]. Heterologous expression of recombinant protein was achieved by treatment with 0.5 mM isopropyl β-D-thiogalactopyranoside for 4 h at 30 °C, followed by affinity chromatography purification using a β-lactose-conjugated Sepharose column. To eliminate contaminating bacterial endotoxins, the proteins were further purified by polymyxin B affinity chromatography (Sigma-Aldrich, St. Louis, MO, USA). The absence of lipopolysaccharide was confirmed using the ToxinSensor Chromogenic LAL Endotoxin Assay Kit (GenScript, Piscataway, NJ, USA). Protein solutions were concentrated by Amicon centrifugal filtration 3K (Millipore, Burlington, MA, USA), dialyzed against PBS containing 10% glycerol and 5 mM β-mercaptoethanol and stored at −80 °C. The integrity of the recombinant protein was monitored by 10% SDS-polyacrylamide gel electrophoresis.

Primary monocytes and THP-1 cells were resuspended in PBS at a concentration of 3 × 10^6^ cells/mL and treated with 100 ng/mL recombinant human TNFα (R&D Systems, Minneapolis, MN, USA) in the presence of 3 µM of human serum albumin (HSA) or recombinant human galectins for 30 min at 37 °C. When specified, galectin-8 was preincubated with 150 mM lactose for 30 min at 37 °C. Cell viability following treatment with recombinant galectins was greater than 93% as assessed using trypan blue dye exclusion. Pull-down experiments and cell aggregation assays were performed with rhGal8 and confirmed using an alternative source of galectin-8 purchased from Sino Biological.

### 2.3. Biotinylation of Cell Surface Proteins

To determine the presence of glycan epitopes on primary monocytes and THP-1 cells, cell surface proteins on apical cell membranes were biotinylated using the Cell Surface Protein Isolation kit (Thermo Fisher Scientific, Waltham, MA, USA) according to the manufacturer’s instructions. The biotinylation efficiency was confirmed by Western blotting using the Avidin-Biotin Complex Kit (Vector Laboratories, Newark, CA, USA).

### 2.4. Lectin Array

Cell surface glycans were assessed using a lectin array (GA-Lectin-70; RayBiotech Life, Peachtree Corners, GA, USA). This technology uses a glass slide spotted with 14 wells, each containing 70 different lectins in duplicate. The specific recognition pattern of each lectin and location within the array is summarized in Supplementary Table S1. All steps were carried out at room temperature under gentle shaking. In these experiments, biotinylated cell lysates from primary human monocytes were dialyzed against PBS at 4 °C overnight. Then, the dialyzed protein (170 μg/mL) was incubated with an equilibrated array slide at 4 °C overnight according to the manufacturer’s instructions. The array was washed under gentle shaking and labeled with Cy3-streptavidin at room temperature for 1 h. The slide was washed, dried, and scanned at 450 nm by RayBiotech. The mean fluorescence intensity for each duplicate lectin spot was averaged and normalized to the untreated monocyte control.

### 2.5. Jacalin Pull-Down and Biotin Blotting

Biotinylated primary monocytes or THP-1 cells (1 × 10^7^ cells/100 μL) were lysed in Triton X-100 buffer (1% Triton X-100, 50 mM Tris base pH 8.0, 150 mM NaCl) containing 1 mM PMSF and a protease inhibitor cocktail (Roche Applied Science, Penzberg, Germany) for 1 h at 4 °C [14]. The insoluble material was cleared by high-speed centrifugation for 30 min at 4 °C. Jacalin agarose beads (Thermo Fisher Scientific, Waltham, MA, USA) were prewashed three times with lysis buffer containing 2% BSA and three times with lysis buffer. Cell lysates (2 mg/mL) were then added to 100 μL of Jacalin agarose beads. The mixture was incubated in a circular shaker (1400 rpm) for 4 h at 4 °C, washed three times in lysis buffer containing 2% BSA, and three times in lysis buffer. The beads were subsequently diluted in 4X Laemmli sample buffer containing 10 mM β-mercaptoethanol and boiled for 5 min at 100 °C. The proteins were run on a 10% SDS-polyacrylamide gel, transferred to PVDF membranes (Bio-Rad Laboratories, Hercules, CA, USA), and blocked with TBST containing 5% nonfat milk. Biotin was detected using the Avidin-Biotin Complex Kit. Positive signal was visualized by chemiluminescence using SuperSignal West Pico Chemiluminescent Substrate (Thermo Fisher Scientific, Waltham, MA, USA).

### 2.6. Flow Cytometry

Human primary monocytes (1 × 10^6^ cells) were resuspended in 100 μL PBS containing 2% fetal bovine serum and incubated with fluorescein-conjugated Jacalin (5 µg/mL, Vector Laboratories) for 30 min at 4 °C. The cells were washed three times before analysis of the fluorescence intensity with a BD™ LSR II flow cytometer (BD Biosciences, San Jose, CA, USA). The geometric mean fluorescence intensity was calculated using FlowJo v10 software (BD Biosciences, San Jose, CA, USA).

### 2.7. Statistical Analysis

Statistical analysis was performed using GraphPad Prism version 9.

## 3. Results and Discussion

### 3.1. Galectins Differentially Regulate the Surface Glycosylation of Human Monocytes

**Figure 1:**
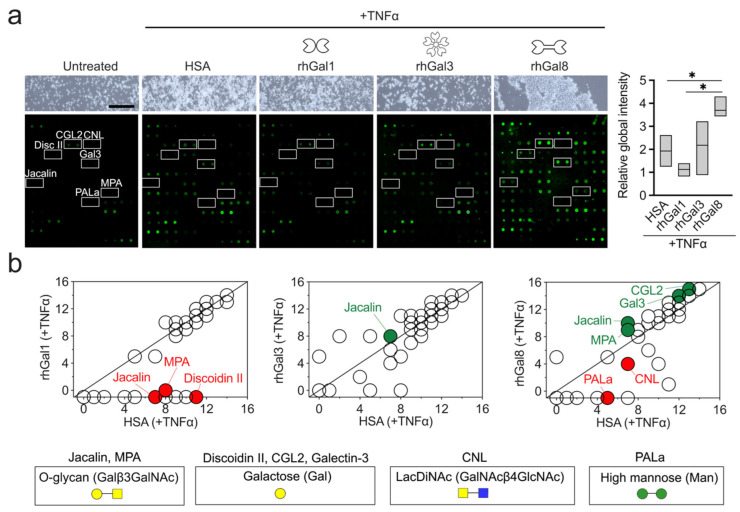
Galectins differentially regulate the surface glycosylation of human monocytes. (**a**) Human primary CD14^+^CD16^−^ monocytes were treated with TNFα in the presence of human serum albumin (HSA) or recombinant human galectins (rhGal1, rhGal3, rhGal8) for 30 min. Untreated monocytes were used as control. Cell surface proteins in each condition were isolated and probed by lectin array (n = 3 independent donors). The data are presented as floating bars (min. to max.) with the line at the median. Significance was determined using one-way ANOVA with Greenhouse–Geisser correction. Scale bar, 250 µm. (**b**) Scatterplot comparing the binding signals derived from images in (**a**). Data were normalized to untreated monocytes. The green and red dots indicate a statistically significant increase or decrease in binding intensity. Significance was determined using the ratio of paired t-test. *, *p* < 0.05.

In this study, we evaluated the ability of three members of the galectin family, representing the prototypical, chimeric, and tandem-repeat types, to direct the expression of glycan epitopes on the cell surface of human monocytes. This is of great importance to the tumor immunology field since the vast majority of membrane and secreted proteins, including immune receptors and ligands, are modified by glycosylation, and galectins have been shown to act as multifunctional mediators of tumor progression [15,16]. We focused on classical CD14^+^CD16^−^ monocytes since they are the most numerous class of monocytic cells and can respond to inflammatory stimuli [17]. These cells were isolated from healthy donor volunteers and subsequently treated with TNFα, one of the first cytokines to appear in the blood after injury and able to trigger monocyte activation [18]. Our initial observation was that galectins differentially regulated the ability of the monocytes to aggregate (Figure 1a). It has been previously reported that monocytes can aggregate spontaneously or under the influence of mediators such as TNFα [19,20]. We found that the presence of galecin-8 in the culture medium exacerbated the formation of cellular aggregates in comparison to TNFα, with or without galectin-1 or -3.

Next, we evaluated changes in cell surface glycosylation using a panel of immobilized lectins recognizing a broad range of glycan structures (Supplementary Table S1). In these experiments, the cell surface protein of human primary monocytes was biotin-tagged to ensure that changes in glycosylation were ascribed to the glycocalyx. Analysis of the relative global intensity revealed that treatment with galectin-8 was highly efficient in stimulating surface glycosylation in these cells (Figure 1a). A detailed analysis of the lectin binding pattern in the array revealed that each galectin differently affected the glycan composition of the monocytic cell surface. Galectin-1 led to a significant reduction in the expression of glycans recognized by Jacalin, MPA and Discoidin II (Figure 1b). On the other hand, treatment with galectin-3 stimulated the binding to Jacalin. Galectin-8 not only enhanced the expression of glycans recognized by Jacalin but also MPA, CGL2 and galectin-3. Remarkably, all of these lectins are associated with the recognition of galactose residues on glycoconjugates. Jacalin and MPA are very close structurally, and both recognize the Thomsen–Friedenreich antigen on mucin-type O-glycans (Galβ1-3GalNAcα-O-Ser/Thr) [21]. Discoidin II is a galactose-binding lectin [22], whereas CGL2 and galectin-3 are two prototype lectins, also closely related, with affinity towards β-galactosides [23].

These experiments also revealed that galectin-8 downregulates the expression of glycans associated with PALa and CNL binding. PALa is a lectin with affinity towards high mannose, while CNL recognizes GalNAcβ4GlcNAc (N,N′-diacetyllactosediamine or LacdiNAc) [24,25]. The LacdiNAc epitope can be found on glycoproteins carrying N- and O-glycans and has been implicated in the regulation of several biological phenomena, such as cell differentiation in human cancer cells [26].

### 3.2. Galectin-8 Induces Jacalin Binding to Human Monocytes

**Figure 2:**
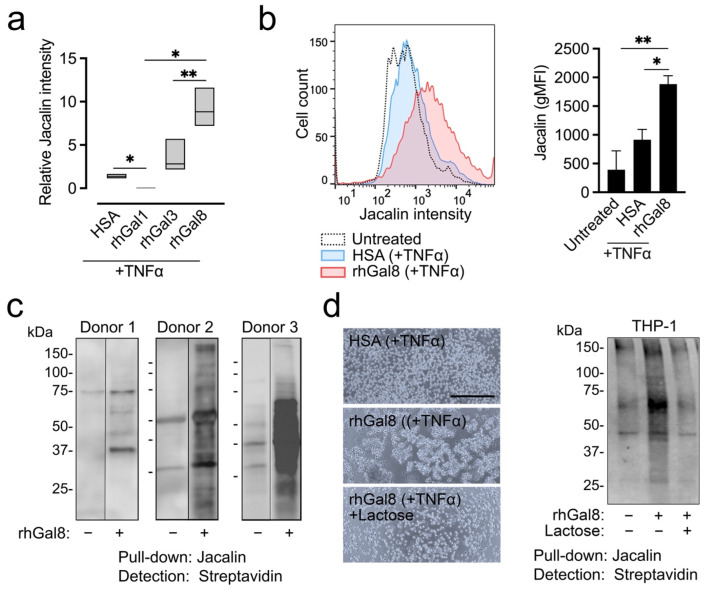
Galectin-8 induces Jacalin binding to human monocytes. (**a**) Human primary CD14^+^CD16^−^ monocytes were treated with TNFα in the presence of human serum albumin (HSA) or recombinant human galectins (rhGal1, rhGal3, rhGal8) for 30 min. Untreated monocytes were used for fluorescence intensity normalization. Binding signals were derived from data in Figure 1a. The data are presented as floating bars (min. to max.) with the line at the median. Significance was determined using one-way ANOVA with Greenhouse–Geisser correction. (**b**) Flow cytometry analysis of human primary monocytes stained with fluorescein-conjugated Jacalin (n = 3 independent donors). The data represent the mean ± SD. Significance was determined using one-way ANOVA with Greenhouse–Geisser correction. (**c**) Cell surface proteins on monocytes isolated from three human donors were labeled with biotin. The cell lysates were normalized for total protein and subjected to Jacalin pull-down. Membranes were probed with streptavidin. (**d**) THP-1 monocytes were processed as indicated in (**c**). Experiments to determine carbohydrate dependence were carried out by preincubation of rhGal8 with 150 mM lactose. Scale bar, 250 µm. *, *p* < 0.05; **, *p* < 0.01.

One of the most dramatic findings in our study was the alteration of Jacalin binding by the tandem-repeat galectin-8. Quantification of the relative amount of Jacalin staining in monocytes revealed that galectin-8 was significantly superior to other galectins in promoting mucin-type O-glycosylation, one of the most abundant and complex posttranslational modifications of proteins (Figure 2a). To confirm this finding, we measured the amount of fluorescein-conjugated Jacalin on the surface of primary monocytes using flow cytometry. Consistent with the lectin array, the addition of recombinant human galectin-8 to the cell culture media led to a significant increase in Jacalin staining compared to TNFα control or untreated cells (Figure 2b). Jacalin pull-down and biotin blotting confirmed the increase in the amount of cell surface protein carrying Jacalin epitopes in monocytes isolated from three human donors, as well as in the monocytic THP-1 cell line, following the addition of exogenous galectin-8 (Figure 2c,d). Additionally, we found in these experiments that the increase in Jacalin epitopes and the ability of galectin-8 to aggregate monocytes were impaired when the lectin was preincubated with lactose, confirming the carbohydrate dependence of this process.

Glycosylation is one of the most highly regulated pathways in the immune system. Alterations in the glycome have been associated with both homeostatic and disease mechanisms of immune cell functions, including immune cell trafficking [27]. Studies in mice have demonstrated that failure to initiate mucin-type O-glycosylation leads to severe impairment of leukocyte recruitment [28]. Of interest are findings using genome-wide association indicating that GALNT4, an enzyme responsible for initiating mucin-type O-glycosylation, plays an instrumental role in the susceptibility to cardiovascular disease [29]. Recent data have evidenced that increased GALNT4 expression in monocytes is sufficient to prime adhesion and transmigration by regulating the O-glycosylation of the P-selectin ligand PSGL-1 in atherosclerosis, a major underlying cause of cardiovascular disease [7]. We hypothesize that an environment rich in galectin-8 could potentially enhance the trafficking of monocytes to areas of tissue damage by regulating the expression of these enzymes. These results might also be relevant to tumor progression since there is indication that the malignancy of human colon cancers and the degree of differentiation of lung squamous cell carcinomas and neuro-endocrine tumors correlate with the expression of galectin-8 [30].

In summary, it is well established that galectins efficiently bind to glycosylated cell surface receptors on immune cells to regulate their activity [12]. Our results provide additional evidence indicating that galectin binding to cell surface receptors also has implications for the character of the glycocalyx in human monocytes. Characterizing the galectin signature in the microenvironment and its influence on the cellular glycome should provide the basis for further studies into the biological activities of monocytes during inflammation.

## Data Availability

The data that support the findings of this study are available from the corresponding author upon request.

## Supplementary Materials

The following are available online at https://www.mdpi.com/article/10.3390/biom12091168/s1, Table S1: Average fluorescence intensity of lectin binding to cell surface proteins isolated from human primary monocytes.
