# Supplementary material for: Galectins Differentially Regulate the Surface Glycosylation of Human Monocytes
Source: Biomolecules. 2022 Aug 23;12(9):1168. doi: 10.3390/biom12091168 (PMC9496102; doi:10.3390/biom12091168)
Supplement: Supplementary file 1 [file biomolecules-12-01168-s001.zip › biomolecules-1756695-supplementary.pdf]

Supplementary Table S1. Average fluorescence intensity of lectin binding to cell surface proteins isolated from human primary monocytes. Cells were treated with TNFα in the presence of human serum albumin (HSA) or recombinant human galectins (rhGal1, rhGal3, rhGal8). Significance was determined against results from cells treated with HSA. Statistically significant data are highlighted in bold.

				+TNFα						
				**HSA**	**rhGal1**		**rhGal3**		**rhGal8**	
Lectin	***Symbol***	***Position***	***Glycan specificity***	***Avg.***	***Avg.***	***p***	***Avg.***	***p***	***Avg.***	***p***
O-glycans										
Amaranthus caudatus	ACL	2CD	Galβ3GalNAc	1.5	0.0	0.2	0.0	0.2	1.1	0.6
Bauhinia purpurea	BPA	3AB	Galβ3GalNAc	1.0	0.0	0.4	0.0	0.4	0.7	0.8
Griffonia simplicifolia I	GS-I	6GH	αGal, α3GalNAc	0.0	0.0	N/A	0.0	N/A	0.0	N/A
Jacalin	Jacalin	7AB	Galβ3GalNAc	2.1	0.0	**<0.001**	2.5	**0.02**	3.0	**<0.001**
Maclura pomifera	MPA	8IJ	Galβ3GalNAc	2.2	0.0	**<0.001**	0.6	0.1	2.6	**0.04**
Peanut	PNA	10CD	Galβ3GalNAc	3.0	0.0	0.2	0.8	0.6	3.0	0.5
Psophocarpus	PTL	10KL	GalNAc, Gal	3.3	3.2	0.4	3.3	0.6	3.5	0.2
Sambucus nigra II	SNA-II	11KL	GalNAc > Gal	0.0	0.0	N/A	0.0	N/A	0.0	N/A
Sophora japonica	SJA	11GH	βGalNAc	3.0	2.7	0.9	2.4	0.2	3.2	0.2
Soybean	SBA	11EF	α > βGalNAc	0.6	0.0	0.4	0.9	0.4	0.0	0.4
Vicia villosa	VVA	12KL	GalNAc	3.3	3.2	0.4	3.2	0.5	1.1	0.1
Wisteria floribunda	WFA	13AB	GalNAc	2.8	2.3	0.5	2.5	0.3	3.3	0.1
N-glycans										
Concanavalin A	Con A	3IJ	αMan, αGlc	3.5	3.6	0.4	3.9	0.3	4.0	0.1
Dolichos biflorus	DBA	3KL	αGalNAc	0.0	0.0	N/A	0.0	N/A	0.6	0.4
Human malectin	Malectin	8EF	Glc2-N-biose	3.6	3.5	0.8	3.7	0.6	3.8	0.5
Leucoagglutinin	PHA-L	9KL	Galβ4GlcNAcβ6(GlcNAc β2Manα3)Manα3	2.3	3.50	0.4	3.0	0.4	1.3	0.5
Phaseolus vulgaris	PHA-P	10AB	Triantenary, tetraantenary structures	2.4	3.3	0.5	3.2	0.5	2.4	0.9

Phaseolus vulgaris Erythroagglutinin	PHA-E	9IJ	Galβ4GlcNAcβ2Manα6(GlcNAcβ4)(GlcNAcβ4Manα3)Manβ4	2.5	3.5	0.5	3.9	0.3	1.4	0.5
Poly-N-acetyllactosamine										
Clitocybe nebularis	CNL	3GH	α/βGalNAc, GalNAcβ4GlcNAc	2.1	0.6	0.1	0.6	0.1	0.6	**0.007**
Erythrina crista-galli	ECA	4GH	Galβ4GlcNAc	0.9	0.0	0.4	0.8	0.4	0.0	0.4
Human galectin1	GAL1	5AB	Branched LacNAc, Gal	2.6	1.9	0.5	3.1	0.2	3.0	0.4
Human galectin1-S	GAL1-S	5CD	Branched LacNAc	2.6	2.8	0.3	2.7	0.7	3.0	0.4
Human galectin2	GAL2	5EF	Branched LacNAc	0.0	0.0	N/A	0.0	N/A	0.0	N/A
Human galectin3	GAL3	5GH	poly LacNAc	3.6	3.5	0.2	3.6	0.9	4.2	**0.04**
Human galectin3C-S	GAL3C-S	5IJ	poly LacNAc	3.1	3.0	0.4	3.2	0.9	3.4	0.6
Human galectin7-S	GAL7-S	5KL	Galβ1-3GlcNAc	2.8	3.0	0.7	3.1	0.6	3.3	0.3
Human galectin9	GAL9	6AB	poly LacNAc	3.6	3.7	0.7	3.7	0.8	3.8	0.7
Laetiporus sulphureus	LSL-N	8AB	LacNAc	3.6	3.7	0.3	3.7	0.7	3.7	0.7
Fucose										
Anguilla anguilla	AAA	1IJ	αFuc	0.0	0.0	N/A	0.0	N/A	0.0	0.0
Aleuria aurantia	AAL	1KL	Fucα6GlcNAc	3.3	3.2	0.5	3.6	0.2	3.1	0.4
Burkholderia cenocepacia	BC2LCN	2KL	Fucα1-2Galβ1-3GalNAc/GlcNAc	2.7	2.9	0.4	3.0	0.6	3.1	0.05
Lotus tetragonolobus	Lotus	7KL	αFuc	1.9	1.6	0.8	2.8	0.4	2.8	0.4
Pseudomonas aeruginosa	PA-IIL	9CD	Fucose, Mannose	0.0	0.0	N/A	0.0	N/A	0.0	N/A
Ralstonia solanacearum	RS-FUC	11AB	Fucose	3.1	3.3	0.6	3.3	0.1	3.3	0.5
Ulex europaeus I	UEA I	12EF	αFuc	2.0	1.07	0.7	1.8	0.8	0.6	0.2
Sialic acid										
Agrocybe cylindracea	ACG	2AB	α2-3 Sialic Acid	3.3	3.4	0.9	3.6	0.6	3.8	0.5
Maackia amurensis I	MAA	8CD	Neu5Acα2-3Gal(β1–4)GlcNAc/Glc	3.0	2. 7	0.3	2.8	0.6	3.3	0.7
Polyporus squamosus	PSL1A	10IJ	α2-6 Sialic Acid	4.2	4.1	0.6	4.3	0.7	4.3	0.8
Sambucus nigra I	SNA-I	11IJ	Neu5Acα2-6Gal, GalNAc	2.9	3.0	0.8	3.1	0.6	3.1	0.7

Sambucus sieboldiana	SAMB	11CD	NeuAcα2-6Gal, GalNAc	3.9	3.9	0.7	4.0	0.6	4.0	0.7
Wheat germ	WGA	13CD	GlcNAc, Sialic acid	2.8	2.8	0.7	2.5	0.6	3.3	0.1
Mannose										
Allium sativum	ASA	2EF	αMan	3.2	3.4	0.4	3.4	0.5	3.5	0.2
Burkholderia cenocepacia	BC2L-A	2IJ	High mannose	2.7	2.9	0.4	2.8	0.8	3.3	0.3
Calystegia sepium	CALSEPA	3CD	High mannose	3.3	3.5	0.2	3.4	0.9	3.8	0.4
Galanthus nivalis	GNA	6CD	αMan	3.5	3.7	0.2	3.7	0.5	3.9	0.3
Griffithia sp.	GRFT	6EF	High mannose	3.3	3.5	0.3	3.6	0.4	3.8	0.2
Hippeastrum hybrid	HHA	6KL	αMan	3.3	3.6	0.1	3.6	0.5	3.7	0.2
Narcissus pseudonarcissus	NPA	8KL	αMan	3.4	3.5	0.7	3.6	0.6	3.7	0.3
Oryza sative	ORYSATA	9AB	High mannose	1.7	0.9	0.5	2.1	0.8	2.2	0.4
Phlebodium aureum	PALa	9GH	High mannose	1.5	0.9	0.4	1.6	0.9	0.0	**0.02**
Vicia faba	VFA	12IJ	αMan	0.9	0.0	0.1	0.3	0.4	0.0	0.1
Mannose/Glucose										
Lens Culinaris	LcH A	7EF	Man, αGlc	3.4	3.5	0.5	3.7	0.3	3.8	0.3
Lentil	LENTIL	7IJ	D-Mannose, D-glucose	3.4	3.4	0.5	3.6	0.5	3.6	0.5
Musa acuminata	BANLEC	2GH	Mannose, Glucose	3.7	3.9	0.5	3.9	0.4	4.1	0.1
Pisum sativum	Pea	10GH	αMan, αGlc	3.5	3.5	0.7	3.7	0.4	3.7	0.5
N-acetylglucosamine										
Datura stramonium	DSA	4EF	GlcNAc	3.2	3.5	0.4	3.4	0.3	3.3	0.8
E. coli	F17AG	4KL	GlcNAc	3.2	3.4	0.5	3.5	0.6	3.7	0.3
Griffonia (Brandeiraea)	GS-II	6IJ	α/βGlcNAc	0.0	0.0	N/A	0.0	N/A	0.0	N/A
Lycopersicon esculentum	LEA	7GH	GlcNAc	1.0	0.0	0.4	0.0	0.4	1.0	0.9
Solanum tuberosum	STL	12AB	GlcNAc	4.0	4.0	0.5	4.1	0.9	4.3	0.5
Ulex europaeus II	UEA-II	12GH	Poly β(1,4)GlcNAc	0.7	0.0	0.4	0.0	0.4	0.0	0.4
Urtica dioica	UDA	12CD	GlcNAc	3.4	3.4	0.7	3.4	0.9	3.61	0.5
Galactose										
Coprinopsis cinerea	CGL2	3EF	βGal	3.8	3.9	0.8	4.0	0.6	4.5	**0.03**

Dictyostelium discoideum I	Discoidin I	4AB	αGalNAc	1.0	0.0	0.4	1.4	0.6	1.2	0.8
Dictyostelium discoideum II	Discoidin II	4CD	Gal, LacNAc	3.0	0.0	**0.01**	2.1	0.2	3.5	0.3
Eunonymus europaeus	EEL	4IJ	Galα3Gal	1.8	2.0	0.9	2.2	0.8	1.9	0.5
Marasmius oreades	MOA	8GH	Galα1-3Galβ1-4GlcNAc, Galα1-3Gal	2.9	1.8	0.3	2.3	0.6	2.2	0.6
Phaseolus lunatus	LBA	7CD	GalNAcα(1,3)[αFuc(1,2)] Gal	0.2	0.0	0.4	0.0	0.4	0.0	0.4
Pleurocybella porrigens	PPL	10EF	α/βGalNAc	0.0	0.0	N/A	0.0	N/A	0.0	N/A
Pseudomonas aeruginosa	PA-IL	9EF	Galα1-3(4)Gal	0.0	0.0	N/A	0.7	0.4	0.0	N/A

N/A, not applicable.
